# A mechanistic study of bisphenol a exposure on asthma through integrating clinical research, network toxicology, machine learning, and multi-omics

**DOI:** 10.3389/fphar.2026.1861505

**Published:** 2026-06-03

**Authors:** Ge Gao, Yuqiu Hao, Lin Zhang, Wei Li, Peng Gao, Yuxi Jia

**Affiliations:** 1 Department of Respiratory and Critical Care Medicine, The Second Affiliated Hospital of Jilin University, Changchun, China; 2 Department of the Second Hospital of Jilin University, Changchun, China

**Keywords:** Asthma, bisphenol A, environmental health, machine learning, network toxicology

## Abstract

**Introduction:**

BPA exposure is known to be correlated with asthma incidence. This study aimed to investigate the molecular mechanisms underlying bisphenol A (BPA)-induced asthma.

**Methods:**

In this study, network toxicology, machine learning, and bioinformatics approaches were integrated with differential expression analysis across multiple datasets. Clinical validation was performed using serum BPA measurement, lung function tests, and quantitative PCR in peripheral blood mononuclear cells.

**Results:**

BPA was significantly elevated in the serum of patients with asthma, showing negative correlations with lung function parameters, such as forced expiratory volume in one second (FEV1), the ratio of FEV1 to forced vital capacity (FEV1/FVC), maximal expiratory flow at 75% of FVC (MEF75), and maximal expiratory flow at 25% of FVC (MEF25), while displaying positive correlations with fractional exhaled nitric oxide, eosinophil count, and eosinophil percentage (p < 0.05). We identified 116 potential target genes associated with BPA-induced asthma, with significant enrichment in the JAK-STAT and Chemokine signaling pathways. Using machine learning and Cytoscape analysis, we further narrowed these to 10 core regulatory genes. BPA exhibits strong binding affinity for five target proteins (CCR3, IL2RB, IL2RA, CXCR4, and CCR7). Quantitative PCR in human peripheral blood mononuclear cells confirmed significant upregulation of the expression of corresponding genes (p < 0.05), with partial gene upregulation correlated with serum BPA levels.

**Discussion:**

BPA exposure affects lung function and systemic immune-inflammatory responses in asthma, potentially modulating asthma pathogenesis by targeting specific genes and the JAK-STAT signaling pathway. These findings provide preliminary evidence for further exploration of the molecular mechanisms and potential therapeutic targets in BPA-induced asthma.

## 1 Introduction

Asthma, a chronic inflammatory disease of the airways, is characterized by episodic airway hyperresponsiveness and reversible airway obstruction. Asthma development is closely associated with the infiltration of various inflammatory cells into the airways, including eosinophils, neutrophils, T lymphocytes (e.g., Th1, Th2, and Th17 subsets), and macrophages. Upon activation, these cells release a series of inflammatory mediators, such as IL-4, IL-5, IL-13, and IgE, thereby inducing airway allergic reactions and bronchoconstriction (Olsthoorn et al., 2025). The global prevalence of asthma reaches as high as 16%, with approximately 55.3% of patients failing to achieve effective control of their condition, imposing a heavy disease burden on families and society (Czira et al., 2022). In addition to traditional risk factors, such as smoking, air pollution, and atopy, exposure to environmental chemicals is considered a significant trigger for the onset and exacerbation of asthma (Mattila et al., 2021). A recent study found that the environmental pollutant HOCl significantly exacerbated airway inflammation and reduced gut microbial diversity in asthmatic mice, effects that were alleviated by taurine supplementation (Chen et al., 2025). In addition, exposure to phthalates, parabens, and other phenols has been linked to significantly higher asthma incidence and severity (Foong et al., 2023; Miller et al., 2025). Among these, bisphenol A (BPA), a widely prevalent environmental endocrine disruptor (EDC), has been implicated in multiple studies as potentially increasing asthma risk, exacerbating symptoms, and triggering other respiratory issues (Abellan et al., 2022). Urban centers with higher BPA exposure exhibit significantly higher asthma rates, highlighting BPA as a potential contributor to the urban asthma burden (Lee et al., 2023).

BPA is an organic synthetic compound frequently used in the production of plastics and resins. It is widely employed in electronic products, automobiles, water bottles, and food packaging (Hahladakis et al., 2023). Diet is considered the most significant source of human exposure to BPA, primarily from food containers, such as canned goods and beverage bottles (EFSA Panel on Food Contact Materials et al., 2023; Wang X. et al., 2023). In 2023, the EFSA reassessed the risks posed by BPA in food to public health, further lowering the Tolerable Daily Intake to 0.2 ng/kg body weight/day (EFSA Panel on Food Contact Materials et al., 2023). Furthermore, the widespread use of BPA has resulted in its presence in water, soil, sediments, and air, where it may adversely affect crop growth (Vasiljevic and Harner, 2021; Loganathan et al., 2023; Giri et al., 2024). A recent meta-analysis revealed a significant association between airborne BPA exposure and asthma (Hatem et al., 2025). Therefore, BPA inhalation exposure could potentially affect the human respiratory system.

BPA, as an EDC, may promote obesity through its estrogen-like activity by disrupting lipid metabolism, and obesity is a significant risk factor for asthma (He et al., 2023). Additionally, estrogenic activity may enhance Th2-type inflammatory responses (Vijeyakumaran et al., 2023). BPA may influence asthma pathogenesis by regulating obesity and Th2 inflammation via its estrogenic activity. BPA exposure induces the upregulation of multiple interleukins, including IL-1β, IL-2, IL-4, IL-5, IL-13, IL-17, and IL-33, in various autoimmune, inflammatory, and allergic diseases (As et al., 2015; Wang et al., 2022; Dong et al., 2023; Al-Shami et al., 2025). Among these factors, IL-4, IL-5, IL-13, and IL-33 are key drivers of type 2 asthma, whereas IL-1β and IL-17 play significant roles in non-Th2 asthma. Additionally, BPA exposure can regulate the expression of the CXCL12-CXCR4 axis and chemokines such as CCL3, CCL4, and CCL5 (Hall and Korach, 2013; Mourot-Bousquenaud et al., 2024). BPA can upregulate the Th2-type immune response in asthma by promoting eosinophilic airway inflammation, as evidenced by elevated pro-allergic cytokine levels, such as IL-4, and increased antigen-specific IgE production (Sawai et al., 2003; Yan et al., 2008). Simultaneously, BPA suppresses immune regulatory mechanisms, including reducing the proportion of Tregs and decreasing the production of IFN-γ and IL-10 (Sawai et al., 2003; Yan et al., 2008). In summary, BPA exposure may promote Th2 inflammatory progression in asthma. However, the specific mechanisms by which BPA participates in asthma-related immune inflammation through its estrogenic properties, modulation of inflammatory factors, and regulation of immune cells remain unknown. We integrated network toxicology, machine learning, multi-omics analysis, and clinical validation methods to comprehensively reveal the potential toxicological pathways of BPA.

## 2 Methods

### 2.1 Study participants

A total of 30 participants were enrolled in this study, comprising 20 patients with asthma and 10 healthy controls. All participants were recruited from the Second Hospital of Jilin University (Changchun, China). An asthma diagnosis was established according to the Global Initiative for Asthma guidelines, based on clinical symptoms and confirmed by spirometry. Healthy controls were matched for age, sex, and body mass index (BMI), with no history of respiratory diseases. Exclusion criteria included cancer, recent infections, and other chronic inflammatory conditions. The study protocol was approved by the ethics committee of the hospital, and all participants provided written informed consent.

### 2.2 Liquid chromatography–tandem mass spectrometry (LC-MS/MS) analysis of BPA in human serum

For the LC-MS/MS analysis of BPA in human serum, samples were prepared as follows: an internal standard (BPA-d16, 100 μg/L) was added to each sample, followed by protein precipitation with 1 mL of acetonitrile. The supernatant was collected, concentrated under nitrogen gas in a 50 °C water bath, diluted to 100 μL with 50% methanol solution, and filtered through a 0.22 μm organic-based microporous membrane. Chromatographic separation was performed using a Brownlee SPP C18 column (2.1 mm × 100 mm, 2.7 μm) with a water-acetonitrile gradient elution (containing 0.1% formic acid aqueous solution). The gradient elution program was as follows: 0–1.0 min (30% B), 1.0–3.0 min (30%–95% B), 3.0–4.5 min (95% B), 4.5–5.0 min (95%–30% B), 5.0–6.0 min (30% B). The flow rate was 0.3 mL/min, column temperature was 40 °C, and injection volume was 10 μL.Calibration standards (10, 20, 50, 100, and 200 ng/mL) were run with each batch, and the curve was linear over this range (r^2^ > 0.99). The LOD and LOQ were both 1 ng/mL, estimated from signal-to-noise ratios of the lowest calibrators. Serum was separated within 2 h of collection and stored at −80 °C until analysis. Procedural blanks (BPA-free water processed alongside samples) were included in every batch and consistently showed BPA below the LOD. Glass or BPA-free polypropylene containers and HPLC-grade reagents were used throughout to minimize exogenous BPA contamination.

### 2.3 Identification of potential asthma-related targets

We obtained the chemical structure and SMILES sequence of BPA from the PubChem database (https://pubchem.ncbi.nlm.nih.gov). Potential targets for BPA (restricted to *Homo sapiens*) were identified using the ChEMBL (http://www.ebi.ac.uk/chembl), SwissTargetPrediction (https://www.swisstargetprediction.ch/), and CTD (https://ctdbase.org) databases. Targets were merged, and duplicates were removed.

Asthma-related disease targets were sourced from the Online Mendelian Inheritance in Man (OMIM) (https://omim.org/) and GeneCards databases (https://www.genecards.org/). Targets were merged, and duplicates were excluded to obtain a comprehensive set of potential asthma-associated targets.

### 2.4 Asthma patient GEO database

Two asthma transcriptome datasets (GSE74986 and GSE76262) were selected from the NCBI GEO database (https://www.ncbi.nlm.nih.gov/geo/browse/). The GSE74968 dataset includes bronchoalveolar lavage (BAL) cell samples from 74 patients with moderate-to-severe asthma and 12 healthy controls, profiled using the GPL6480 platform (Agilent-014850 Whole Human Genome Microarray 4x44 K G4112 F). The GSE76262 dataset consists of induced sputum samples from 118 patients with moderate-to-severe asthma (48 male; mean age 51.5 years) and 21 healthy controls (15 male; mean age 37.8 years), profiled using the GPL13158 platform ([HT_HG-U133_Plus_PM] Affymetrix HT HG-U133+ PM Array Plate). A multi-step normalization strategy was employed to mitigate batch effects. Surrogate variable analysis was performed using the corresponding R package to identify and correct for potential confounding factors present in the discovery cohort. Subsequently, residual batch-related variation was further eliminated using the ComBat Harmonization method within a parametric-empirical Bayesian framework. To evaluate the effectiveness of these corrections, we performed a visual inspection of the processed data using principal component analysis (PCA). The harmonized data exhibited improved clustering of samples within batches and more converged distributions across different batches in the low-dimensional space.

### 2.5 Differential gene expression analysis

Transcriptomic data were analyzed using the limma package. Differentially expressed genes (DEGs) were defined as those with a false discovery rate (FDR)-corrected p-value < 0.05 and |log_2_FC| > 0.585 (corresponding to a 1.5-fold expression change). Differential genes were intersected with OMIM and GeneCards database genes to obtain asthma disease pivotal targets, which were visualized using Venn diagrams.

### 2.6 Identification of asthma-related disease targets

The overlap between asthma hub targets and BPA-predicted targets was identified, and the intersecting BPA–asthma targets were visualized using Venn diagrams.

### 2.7 Functional pathway analysis of target genes

BPA–asthma targets were analyzed for Gene Ontology (GO) functional analysis and Kyoto Encyclopedia of Genes and Genomes (KEGG) pathway enrichment analysis using the clusterProfiler software package. Significantly enriched terms were based on a p-value <0.05 and the human genome database org.Hs.eg.db was used as the annotation background.

### 2.8 Construction of protein–protein interaction (PPI) networks

PPI networks were constructed using the STRING database (https://string-db.org/), with *Homo sapiens* as the selected species and an interaction score threshold of ≥0.4. BPA–asthma targets were used as candidate nodes to generate the PPI network maps. Cytoscape software was used for network visualization and topological analysis, and PPI network diagrams were generated. The CytoHubba plugin was used to identify the top 50 important genes using the Maximum Group Centrality (MCC) algorithm and Degree, and the intersection of these results was defined as the set of core protein targets.

### 2.9 Core target selection by machine learning algorithms

Support vector machine recursive feature elimination (SVM-RFE) algorithm was used to screen key genes. Gene expression data were normalized by Z-score and feature weights were calculated using a linear kernel support vector machine (penalty parameter C = 10). In a 10-fold cross-validation framework, feature ordering was determined by 10 random subsamples, and redundant features were recursively eliminated according to their weights (half of the features were eliminated each time for >50 features, and one at a time for ≤50). The optimal number of features was determined by the criterion of minimizing the cross-validation error rate, and the resulting core gene set was intersected with the core protein targets to finally obtain the core BPA-asthma target set. All analyses were performed in R language (4.2.0), and the random seed was set to 12,345 to ensure reproducibility.

### 2.10 Immune infiltration analysis

The CIBERSORT algorithm was used to estimate the proportions of 22 immune cells in the GEO datasets GSE74968 and GSE76262 (LM22 signature, 1,000 permutations; only samples with P < 0.05 retained), and the results were quantitatively normalized. The Wilcoxon rank sum test was used to compare immune cell infiltration levels between groups, while Spearman correlation analysis was used to assess the association between key gene expression and immune cell abundance. The results were visualized using heatmaps and customized graphics.

### 2.11 Quantitative real-time PCR (qPCR)

Total RNA was extracted from peripheral blood mononuclear cells (PBMCs) using TRIzol reagent (samples with concentration <20 ng/μL or A260/A280 outside 1.8–2.1 were excluded). cDNA was synthesized with a reverse transcription kit according to the manufacturer’s instructions. qPCR was performed using SYBR Green Master Mix on a QuantStudio system. Primer sequences for the target genes (CCR3, CCR7, IL2RB, IL2RA, and CXCR4) and the internal control (GAPDH) were designed and validated (Supplementary Table S3). Relative gene expression was calculated using the 2^–ΔΔCt method.

### 2.12 Molecular docking

To investigate BPA interactions with identified core target genes, molecular docking analysis was conducted. The three-dimensional structure of BPA structure was obtained from PubChem, while crystal structures of core target proteins were retrieved from the RCSB PDB database (Supplementary Table S4). Protein structures were processed in PyMOL by removing water molecules and native ligands. Hydrogen atoms were added, and charges were assigned using AutoDock Tools 1.5.6 for protein preparation. Molecular docking was performed with AutoDock Vina. Docking grids were defined at the predicted active sites of each target protein, with box dimensions adjusted according to ligand size. Docking results were visualized in PyMOL for binding modes and key intermolecular interactions.

### 2.13 Statistical analysis

Statistical analyses were performed with R (version 4.2.0). Continuous data were expressed as mean ± standard deviation or median with interquartile range and compared using the Student’s t-test or Mann–Whitney U test. Categorical variables were compared using the chi-squared test. Correlations were assessed using Pearson or Spearman methods based on data distribution. Statistical significance was set at p < 0.05.

## 3 Results

### 3.1 Analysis of serum BPA levels in asthmatic and healthy participants

#### 3.1.1 Comparison of baseline characteristics

A total of 20 patients with asthma and 10 healthy controls were included in this study. No significant differences were observed between the two groups in terms of age, sex, BMI, or smoking history (Table 1).

**TABLE 1:** Baseline characteristics of patients with asthma and healthy individuals.

Variant	Patients with asthma	Healthy controls	P-value
Total (N)	20	10	—
Age, N (years)	47.1 ± 14.7	43.7 ± 13.8	0.447
Men (%)	7 (35.0)	4 (40.0)	0.789
BMI (kg/m^2^)	23.0 ± 4.2	23.0 ± 3.2	0.986
Ex-smokers, N (%)	7 (35.0)	3 (30.0)	0.560
Current smokers, N (%)	3 (15.0)	1 (10.0)	0.593
Smoking index (packets/year)	0 (0,6)	0 (0,6)	0.686
FEV1 predicted	2.8 (2.6, 3.2)	2.7 (2.4, 3.2)	0.495
FVC predicted	3.4 (3.1, 3.7)	3.2 (2.9, 3.6)	0.194
FEV1 (pre)	2.1 ± 0.9	2.8 ± 0.4	0.009
FVC (pre)	3.2 ± 1.0	3.5 ± 0.4	0.227
FEV1/FVC (%) (pre)	64.0 ± 13.2	82.0 ± 4.8	<0.001
MEF75 (pre)	2.9 (1.7, 5.4)	5.8 (5.5, 6.2)	0.004
MEF25 (pre)	0.5 (0.3, 1.1)	1.5 (1.3, 2.0)	<0.001
FEV1 (post)	2.3 ± 0.9	2.9 ± 0.5	0.043
FVC (post)	3.3 ± 0.9	3.6 ± 0.8	0.523
FEV1/FVC (%) (post)	66.8 ± 13.4	86.4 ± 5.7	<0.001
MEF75 (post)	3.9 ± 2.2	6.8 ± 1.4	<0.001
MEF25 (post)	0.5 (0.3, 1.1)	1.6 (0.9, 2.1)	0.002
FeNO (PPb)	28.1 ± 19.3	7.8 ± 1.3	<0.001
Eosinophils (×10^9^/L)	0.4 ± 0.4	0.1 ± 0.1	0.017
Eosinophils (%)	3.4 (1.7, 5.2)	1.5 (1.0, 2.0)	0.009
Neutrophils (×10^9^/L)	4.2 ± 1.5	3.3 ± 0.9	0.088
Neutrophils (%)	58.0 ± 9.3	54.3 ± 5.8	0.255
Monocytes (×10^9^/L)	0.4 ± 0.1	0.4 ± 0.7	0.087
Monocytes (%)	5.8 ± 2.0	6.0 ± 1.0	0.772
Lymphocytes (×10^9^/L)	2.3 ± 0.7	2.3 ± 1.3	0.993
Lymphocytes (%)	30.6 ± 9.2	37.6 ± 6.2	0.041

BMI: body mass index; FEV1: first second expiratory volume with exertion; FVC: forced vital capacity; MEF: maximal expiratory flow. Data are expressed as mean ± standard deviation or median (interquartile spacing) unless otherwise stated.

Significant airflow limitation was observed in patients with asthma (Table 1). Before bronchodilator administration and after 15 min of inhalation, the first second expiratory volume with exertion (FEV1), FEV1/FVC ratio, and maximal expiratory flow rate at 25% and 75% of lung capacity (MEF50 and MEF75) of patients with asthma were significantly lower than those of the healthy group. Additionally, their expired air nitric oxide (FeNO) levels were significantly higher than those of the healthy group, indicating persistent airway obstruction and inflammation.

Peripheral blood eosinophil counts and eosinophil percentage levels were significantly higher in patients with asthma (p < 0.05) than in the controls (Table 1), indicating increased airway inflammation. Additionally, the percentage of lymphocytes was lower in the patients with asthma than in the healthy participants (p < 0.05) even though no significant difference was found in their lymphocyte counts, This confirms the presence of an abnormal immune response in patients with asthma.

#### 3.1.2 Distribution of serum BPA levels and its relationship with clinical characteristics

**FIGURE 1:**
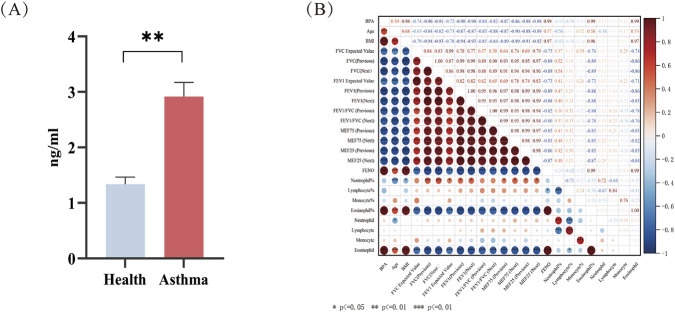
**(A)** Expression levels of bisphenol A in patients with asthma (n = 20) and healthy individuals (n = 10). **(B)** Correlation between bisphenol A and characteristics of patients with asthma.

LC-MS/MS detection tips, serum BPA levels were higher in patients with asthma than in healthy participants (p < 0.05), indicating a potential link between BPA exposure, airway inflammation, and asthma pathophysiology (Figure 1A). Our results are consistent with those from previous studies on serum levels of BPA in asthmatic children and pregnant women (Tang et al., 2022).

As shown in Figure 1B, a significant positive correlation was observed between BPA levels and BMI, suggesting that obesity may promote BPA accumulation via lipid metabolism pathways (Lu et al., 2025). BPA was also significantly negatively correlated with age.

BPA levels showed significant negative correlations with multiple spirometry indices (all p < 0.01), including pre- and post-bronchodilator FVC, FEV_1_, and FEV_1_/FVC ratio. Mid-expiratory flow rates (MEF75 and MEF25) were also negatively correlated with BPA levels (r ranging from −0.82 to −0.87), indicating a potential link between BPA exposure and impaired lung function, particularly small airway dysfunction.

BPA was positively correlated with eosinophil percentage and count but negatively correlated with neutrophil percentage, suggesting that BPA may specifically enhance eosinophilic inflammation—a hallmark of Th2 asthma—by modulating inflammatory cell profiles. Therefore, an in-depth analysis of the molecular mechanisms of BPA effects on asthma from the perspective of network toxicology has important environmental health implications.

### 3.2 Prediction of potential BPA–Asthma targets

A total of 26,531 potential biological targets of BPA were predicted after integrating and removing redundant entries (Supplementary Figure S1A). Meanwhile, a total of 2840 unique asthma-related targets were obtained by searching the GeneCards and OMIM databases and after de-duplication (Supplementary Figure S1B). Venn diagram analysis revealed a total of 2410 common targets, these may serve as potential targets for BPA-induced asthma (Supplementary Figure S1C).

### 3.3 Validation and functional and pathway enrichment analysis of candidate BPA–Asthma targets in the GEO database

**FIGURE 2:**
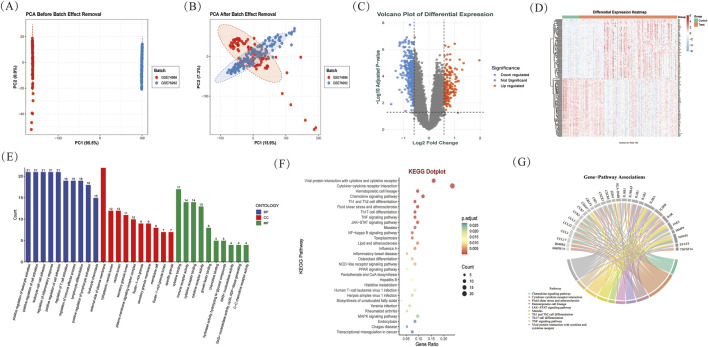
Identification and enrichment analysis of BPA-associated asthma targets using GEO datasets. **(A)** PCA scatterplot showing separation between the GSE74968 and GSE76262 datasets before batch correction, indicating a batch effect. **(B)** PCA scatterplot after batch correction shows GSE74968 and GSE76262 dataset integration, indicating a reduced batch effect **(C)** Volcano plot showing DEGs based on logFC and significance. Red indicates upregulated genes, green indicates downregulated genes, and gray indicates genes with no significant difference **(D)** Heatmap showing DEG expression pattern in each sample. Red indicates upregulation and blue indicates downregulation **(E)** Top 10 functions of BP, CC, and MF analyses **(F)** Bubble plots of KEGG pathway analyses **(G)** Chord plots of the top 10 significantly enriched KEGG pathways (colored arcs) with their shared genes (gray arcs). The width of ribbons indicates the strength of gene-pathway association.

To further identify BPA-induced asthma key targets, this study integrated asthma-related transcriptomic data from GSE74968 and GSE76262, merged the raw data, and performed batch effect correction and normalization. PCA demonstrated that the normalized dataset displayed a clearer trend of sample clustering, indicating effective control of batch effects (Figures 2A,B). Subsequently, 549 genes were identified as significantly differentially expressed between the asthma and control groups, and volcano plots were plotted to visualize all the DEGs. Heatmaps were used to display expression patterns (Figures 2C,D), with the top 25 genes labeled among the top 100 significantly DEGs (Supplementary Figure S2A). Finally, intersecting these asthma DEGs with the 2,410 BPA–asthma potential targets predicted in the previous section yielded 116 candidate genes, which may represent the core targets through which BPA regulates asthma (Supplementary Figure S2B).

We functionally characterized 116 target genes for GO and KEGG enrichment analysis to explore the mechanism of BPA-induced asthma. The top 10 GO terms with the lowest FDR in each category were selected and visualized (Figure 2E; Supplementary Figure S2C), revealing the functions of the core genes at three levels. In terms of biological processes (BP), the core genes were significantly enriched for “positive regulation of leukocyte activation,” “regulation of inflammatory response,” “lymphocyte differentiation,” and “leukocyte chemotaxis,” contributing to the over-activation of immune cells and Th1/Th2/Th17 lymphocyte subpopulation imbalance in asthmatic airways. In terms of cellular components (CC), the genes were mainly localized to “plasma membrane signaling receptor complexes,” “membrane rafts,” and various granules, suggesting their involvement in intercellular communication and release of inflammatory mediators. In terms of molecular function (MF), the significant enrichment of “cytokine binding,” “cytokine receptor activity,” and “chemokine binding” confirmed the role of core genes in asthma through the cytokine-receptor interaction network. KEGG pathway enrichment analysis (Figure 2F) showed that core genes were significantly enriched in classical pathways associated with immunity and inflammation, such as “cytokine-cytokine receptor interaction,” “Chemokine signaling pathway,” and “JAK-STAT signaling pathway,” which coordinate the recruitment and activation of immune cells and may serve as targets for environmental pollutants (e.g., BPA). To visualize the association between core genes and pathways, we constructed a gene-pathway chord diagram (Figure 2G), revealing a dense network of interactions. The “cytokine-cytokine receptor interaction” and “Chemokine signaling pathway” acted as core hubs connecting most genes, suggesting they form a functional module to synergistically regulate asthma-associated immune-inflammatory responses.

### 3.4 Screening and expression patterns of core BPA–Asthma targets

**FIGURE 3:**
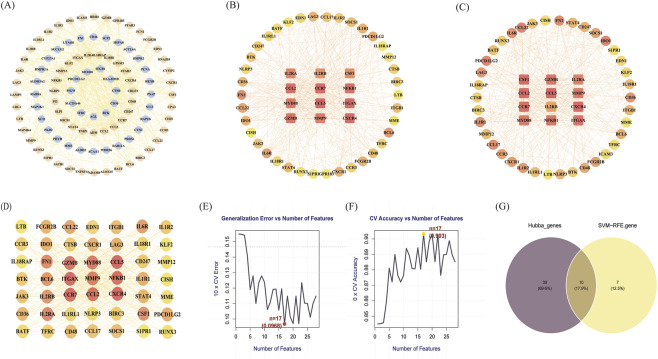
Core target PPI network and machine learning screening of core genes. **(A)** PPI network plot of 116 targets, with upregulated genes in asthma in yellow and downregulated genes in asthma in blue. **(B)** Core targets ranked among the top 50 Hubba genes based on degree value. **(C)** Core targets ranked among the top 50 Hubba genes based on MCC value. **(D)** Intersection of the top 50 Hubba genes and 50 Hubba genes identified by matrix ranking. **(E)** Generalization error of the SVM-RFE model versus the number of features, with the minimal error (0.0968) observed at 17 features. **(F)** Cross-validation accuracy of the SVM-RFE model across different feature sizes. The highest accuracy (0.903) was achieved using 17 features. **(G)** Venn plots showing the 10 core genes shared by the CytoHubba and SVM-RFE methods.

Subsequently, a PPI network of 116 targets (STRING/Cytoscape) identified 49 core targets based on overlapping degree- and MCC-ranked nodes from CytoHubba (Figures 3A–D).

The SVM-RFE analysis was used to identify an optimal subset of features containing 17 genes. A linear kernel was used, and features were recursively eliminated based on weight rankings under 10-fold cross-validation. This 17-gene set achieved the lowest average error rate (0.0968) and highest average accuracy (0.903) across all tested subset sizes (Figures 3E,F). The error rate was substantially lower than the chance baseline of 0.500, indicating that these genes carry genuine discriminative information for asthma status.

Integration of PPI and SVM-RFE analyses identified 10 high-confidence core targets for BPA-induced asthma (Figure 3G).

### 3.5 Analysis of immune infiltration

**FIGURE 4:**
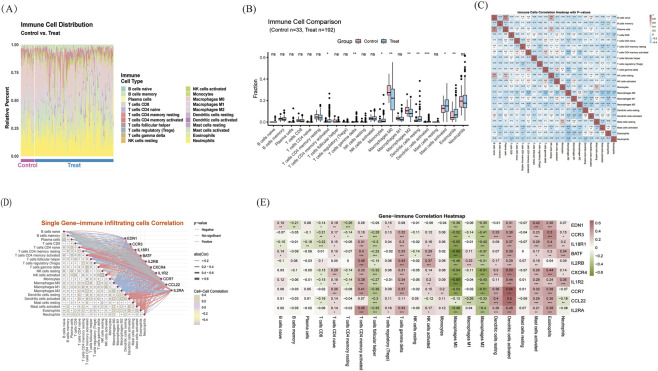
Immune infiltration analysis of the asthma GEO dataset. **(A,B)** CIBERSORT algorithm analysis of 22 different immune cell types in patients with asthma and healthy individuals. **(C)** Correlation matrix of immune cells in patients with asthma. **(D,E)** Correlation between core target genes and immune cell infiltration in asthma.

The relative proportions of monocytes, resting dendritic cells, activated dendritic cells, activated mast cells, and eosinophils,as predicted by CIBERSORT, were significantly higher in the asthma group than in controls, whereas the proportions of M0-and M2-type macrophages were significantly lower, and this discrepancy was mainly reflected in the suppression and activation of the Th1/Th2-associated immune response (Figures 4A,B). Further analyses revealed a network of specific correlations between different immune cell subpopulations (Figure 4C). Notably, plasma cells were significantly positively correlated with resting B cells and resting NK cells, whereas activated CD4 memory T cells were negatively correlated with M0 macrophages.

To explore the functional link between immune infiltration and key molecular drivers, we analyzed the correlation between the expression levels of 10 core target genes and the immune cell infiltration profiles (Figures 4D,E). Expression of EDN1, CCR3, IL18R1, BATF, IL2RB, CXCR4, IL1R2, CCR7, CCL22, and IL2RA was significantly negatively correlated with M0 and M2 macrophages but positively correlated with activated dendritic cells and mast cells. All genes except IL2RB were positively associated with resting CD4^+^ T cells and eosinophils. These findings reveal that the above genes are closely involved in airway remodeling, acute allergic inflammatory response, and overactivation of Th2-dominant immune response by regulating complex immune networks in asthma.

### 3.6 Molecular docking and clinical validation of BPA with core targets

**FIGURE 5:**
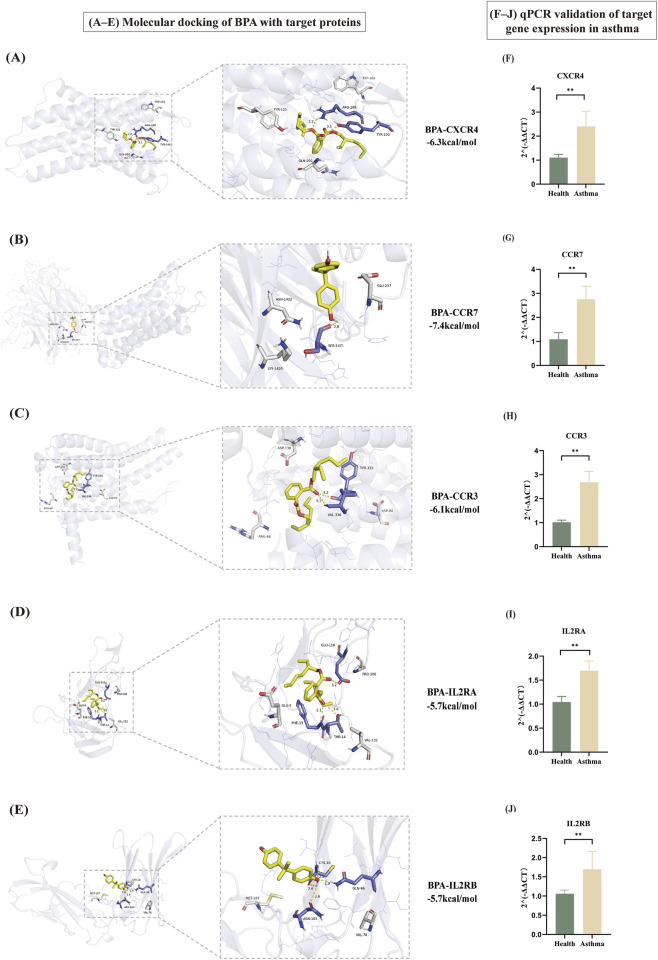
Molecular docking and clinical validation of core targets. **(A–E)** Molecular docking of BPA with core target proteins. **(B–J)** Expression of CXCR4, CCR7, CCR3, IL2RA, and IL2RB in peripheral blood mononuclear cells from patients with asthma (n = 15) and healthy individuals (n = 9).

BATF and CCL22 BATF and CCL22 lacked PDB structures. BPA showed predicted favorable binding energies to five target proteins, with AutoDock scores below −5 kcal/mol, suggesting potential binding. Docking conformations (Figures 5A–E) showed the predicted binding poses for all complexes.

To further clarify the potential role of the above target genes in asthma, peripheral blood samples were collected and PBMCs were isolated. The qPCR subset (15 asthma, 9 controls) was comparable to the full cohort in key characteristics (Supplementary Tables S1, S2). RT-qPCR analysis of core genes showed significantly higher mRNA expression in the asthma group than in the control group, indicating a positive association with asthma risk (Figures 5F–J). Subsequently, we performed the Spearman’s analysis of mRNA levels of core genes of PBMCs and serum BPA levels in patients with asthma, and confirmed the correlation between mRNA expression of CCR3, IL2RA, CXCR4, and CCR7, and BPA (Table 2).

**TABLE 2:** Correlation between serum BPA concentration and expression levels of candidate genes.

Gene	Correlation coefficient (r)	P-value
IL2RA	0.532	0.041
IL2RB	0.486	0.066
CXCR4	0.561	0.03
CCR3	0.525	0.044
CCR7	0.561	0.03

Correlation was assessed using Spearman’s rank correlation analysis. Genes are listed in descending order of the correlation coefficient. (n = 15).

## 4 Discussion

This study integrated network toxicology, machine learning, multi-omics analysis, and clinical validation to systematically explore the potential mechanism of BPA in asthma. Five core genes, CCR3, IL2RB, IL2RA, CXCR4, and CCR7, were identified and significantly upregulated in peripheral blood of patients with asthma, which was confirmed by qPCR.

Serum BPA levels are significantly elevated in patients with asthma and are positively correlated with BMI, a result consistent with the fat-soluble nature of BPA. Meanwhile, the level of BPA exposure was significantly negatively correlated with several lung function indices, including FEV_1_, FVC, FEV_1_/FVC ratio, MEF25, and MEF75 measured before and after salbutamol administration. This suggests that BPA may play an important role in the pathophysiological process of asthma by exacerbating airway inflammation and remodeling and impairing airway function, including small airways. Additionally, BPA levels were positively correlated with peripheral blood eosinophil counts and percentages but negatively correlated with lymphocyte counts. Elevated eosinophils, as key effector cells of Th2-type inflammation, are usually accompanied by the release of cytokines such as IL-5 and IL-13, which are directly involved in airway hyperresponsiveness and inflammatory responses, while a decreased percentage of lymphocytes may reflect an imbalance in immunoregulation.

Using network toxicology and bioinformatics analyses, we identified 116 BPA–asthma co-targets. GO and KEGG enrichment analyses indicated that BPA exposure may affect asthma development primarily through immuno-inflammatory pathways, including lymphocyte differentiation, the JAK-STAT signaling pathway, and the chemokine signaling pathway.

BPA regulates primary human lymphocytes in a concentration-dependent manner. At high concentrations (250 μM), BPA significantly inhibits lymphocyte proliferation and function, alters cytokine levels, and interferes with immune signaling pathways (Zhang et al., 2024). In animal and *in vitro* models, BPA promotes Th2 cell differentiation by upregulating GATA-3, enhancing CCL1 expression, and modulating the IL-10/IL-12p70 balance, while also interfering with dendritic cell function (Guo et al., 2010). This Th2 over-differentiation represents a key immunopathological mechanism, suggesting that BPA may exacerbate Th2-type asthma by promoting Th2-biased immune responses (Wang et al., 2022).

BPA can activate the JAK-STAT signaling pathway in cells (Ptak and Gregoraszczuk, 2012). The JAK-STAT pathway is a core inflammatory pathway in the pathogenesis of asthma, and its inhibition is of great significance in asthma treatment (Jung et al., 2025). Among them, STAT3 may be involved in asthma by regulating Th17/Treg homeostasis and promoting M2 macrophage polarization during asthma pathology (Zhong et al., 2023; Tao et al., 2025). JAK1 inhibitors have been approved for the clinical treatment of asthma and have shown promising efficacy in Th2-type asthma (Nilsson et al., 2023).

BPA regulates the immune inflammatory response through chemokine signaling pathways. BPA inhibits CXCL8 secretion in human neutrophils (Peillex et al., 2021) and may inhibit CXCL8 expression in endometrial stromal cells (DSCs) through activation of the ERK pathway (Li et al., 2018). In contrast, in mouse bone marrow-derived dendritic cells, BPA and its substitutes significantly induced a more than two-fold increase in the secretion of CCL3, CCL4, and/or CCL5 (Mourot-Bousquenaud et al., 2024). CXCL8 promotes neutrophil recruitment and is of central pathological importance in airway inflammation in severe asthma (Yuliani et al., 2022). The CCL3-CCR1 axis acts on monocytes, eosinophils, and basophils in asthma T lymphocyte recruitment (Castan et al., 2017). CCL4 promotes eosinophil adhesion to airway epithelium and exacerbates Th2 inflammation by cleaving the N-terminal structural domain of MUC1 protein (Kobayashi et al., 2024). In contrast, high CCL5 expression was associated with a mixed inflammatory phenotype, characterized by simultaneous eosinophilia and neutrophilia, suggesting that CCL5 plays a key role in asthma severity (Gauthier et al., 2023). Furthermore, in ovarian cancer cells, EDCs such as BPA may promote the growth via CXCL12–CXCR4 signaling (Hall and Korach, 2013). BPA has also been implicated in ulcerative colitis through CCR3-mediated eosinophil regulation (Huang et al., 2022). Collectively, these findings suggest that BPA may contribute to asthma-related inflammation through CCR3, CCR7, and CXCR4 pathways.

We further combined machine learning algorithms with PPI network analysis to screen 10 core genes. In this analysis, SVM-RFE was employed for feature selection rather than diagnostic classification, identifying genes that most effectively differentiate asthma from healthy controls. Given that asthma is a chronic inflammatory disease closely related to the immune system, we assessed the infiltration levels of 22 immune cell types in healthy controls versus patients with asthma using the CIBERSORT algorithm on the GSE74968 and GSE76262 transcriptome datasets. We further analyzed the correlations between these 10 core genes and immune cell infiltration. We found that immune cells, including CD4 memory T cells, resting monocytes, activated dendritic cells, activated mast cells, and eosinophils, were significantly elevated in the asthma group. Among these, eosinophils and Th2 cells are the key inflammatory cells in Th2-type asthma. Activated CD4^+^ memory T cells sensitively recognize allergens and rapidly release Th2-type cytokines, such as IL-4, IL-5, and IL-13, playing a key role in acute asthma attacks (Ruterbusch et al., 2020). Additionally, γδ T cells promote the differentiation of initial CD4^+^ T cells toward Th2, thereby amplifying the Th2 inflammatory response and participating in the initiation of early asthma inflammation (Ferrick et al., 1995). Dendritic cells promote Th2 cell differentiation by binding IgE-crosslinked allergens (FcεRI) and via the OX40 L-OX40 signaling pathway (Chen et al., 2013). High FcεRI expression on mast cell surface has an important role in Th2-type asthma pathogenesis (Maurer et al., 2019).

Subsequent analysis of the 10 core targets in relation to immune cell infiltration revealed that the expression of EDN1, CCR3, IL18R1, BATF, IL2RB, CXCR4, IL1R2, CCR7, CCL22, and IL2RA positively correlated with the infiltration scores of activated dendritic cells and mast cells and negatively correlated with the M0 and M2 macrophage infiltration scores. All core targets, except for IL2RB, were positively correlated with eosinophils and resting CD4^+^ T cells. These results suggest that BPA may be involved in the pathogenesis of asthma by upregulating these core targets. The expression of these targets was positively associated with the estimated proportions of activated dendritic cells and mast cells, as well as resting CD4^+^ T cells and eosinophils, which are key cellular drivers of Th2-type responses. The negative association with M2 macrophage proportions further hints that BPA may counteract an anti-inflammatory microenvironment and indirectly contribute to airway inflammation.

Further molecular docking identified five core genes (binding energy less than −5), i.e., CCR3, CCR7, CXCR4, IL2RB, and IL2RA, and verified that these core genes were significantly upregulated in the asthma group by qPCR in PBMCs cells. Subsequently, a correlation between serum BPA levels and mRNA expression of CCR3, IL2RA, CXCR4 and CCR7 in PBMCs from asthmatics was confirmed by Spearman correlation analysis, which supports the hypothesis that BPA may be involved in asthma pathogenesis through these core targets. Although we did not definitively confirm the correlation of IL2RB with BPA in asthma patients, it is possible that this is related to the number of sample sizes.

CCR3, CCR7, and CXCR4 belong to the chemokine receptor family, which plays a central role in directed migration of immune cells, inflammatory response, and immune regulation. CCR3 is primarily expressed in eosinophils, mast cells, Th2 cells, and airway epithelial cells. Upon binding to its high-affinity ligands CCL11 and CCL24, CCR3 activates downstream signaling pathways, such as MAPK (ERK1/2, p38) and PI3K/AKT, thereby regulating eosinophil chemotaxis, activation, and migration, and contributing to Th2-type allergic responses (Méndez-Enríquez et al., 2014). Knockout of the bone marrow CCR3 gene significantly increases Th2 inflammation and IgE expression in asthmatic mice (Dai et al., 2022). In asthma models, platelets are recruited to lung tissue in a CRR3-dependent manner and are involved in asthma allergen sensitization, leukocyte recruitment, bronchospasm, and airway wall remodeling disease processes (Shah et al., 2021). CCR7 is highly expressed in dendritic cells and naive T cells, and, through its ligands CCL19 and CCL21, it directs lymphocyte homing to secondary lymphoid organs and initiates immune responses (Förster et al., 2008). *In vitro* experiments revealed that activation of the CCR7/CCL19 axis may promote migration and accumulation of fibroblasts into the airways, leading to airway remodeling in asthma (Wang et al., 2015). Asthma mouse model studies further confirmed that the CCR7 ligand CCL19 increases STAT5 phosphorylation, induces Th2 cell differentiation and expression of IL-2 signaling pathway-related genes, and promotes asthma progression (Nakano et al., 2024). CXCR4 binding to the ligand CXCL12 induces eosinophil migration and fibroblast chemotaxis in the asthmatic process (Wang et al., 2015). It also activates PKC, MAPK, Src kinase, and PI3K/AKT signaling pathways, which play a role in cell proliferation, migration, and lymphocyte recirculation (Smit et al., 2021). CXCR4-overexpressing neutrophils release neutrophil extracellular traps in response to the lung microenvironment, thereby modulating dendritic cell function and promoting the onset and progression of allergic asthma (Radermecker et al., 2019). Furthermore, in ovarian cancer cells, EDCs such as BPA may promote growth via CXCL12–CXCR4 si suggest that BPA may contribute to asthma-related inflammation through CCR3, CCR7, and CXCR4 pathways.

IL2RA and IL2RB form a high-affinity IL-2 receptor, which is predominantly expressed on activated T cells, especially Tregs (Seddiki et al., 2006). In asthma, impaired function or insufficient numbers of Treg cells disrupt immunosuppressive balance, leading to overactivation of the Th2 response (Palomares et al., 2017). In asthma patients, IL2RA expression is crucial for the survival and proliferation of Th2 cells. Furthermore, IL2RA expression in Th2 cells is upstream-regulated by the dopamine/DRD4/cAMP signaling axis, promoting the differentiation of Th2 cells into tissue-resident memory cells. This ultimately drives the rapid inflammatory response triggered by allergens in asthma (Wang W. et al., 2023). IL2RB serves as a common subunit for IL-2 and IL-15 receptors. Impaired expression and function of mouse IL2RB disrupts STAT5 signaling, leading to sustained T cell activation, abnormal differentiation, and reduced Treg numbers. This cascade of events ultimately causes T cell activation and differentiation abnormalities, triggering immune dysregulation (Cabrera-Martinez et al., 2025). BPA upregulates GATA3 and RORγt expression in Treg cells by antagonizing the ERβ signaling pathway, contributing to the differentiation of Treg cells toward Th2 or Th17 cells and impairing immunosuppression (Fischer et al., 2024). This suggests a role for BPA in asthma via IL2RA and IL2RB regulation.

The clinical sample size is limited, and the small qPCR subset precluded multivariable adjustment for potential confounders such as age and BMI; the observed correlations therefore require validation in larger, independent cohorts. Additionally,the network toxicology screen relied on database predictions and curated associations, which may include false positives; these were narrowed by intersecting with disease genes and DEGs, but the initial target pool should be viewed with caution. The specific functional consequences of BPA interactions with core targets and downstream signaling pathways need further validation by *ex vivo* gain- and loss-of-function experiments. Exploring the combined effects of BPA with other environmental pollutants represents an important direction for future research.

## 5 Conclusion

We systematically explored the potential molecular mechanism by which BPA exposure may promote asthma development through targeted immunomodulation, using integrated multi-omics analysis. Clinical trials confirmed that elevated serum BPA levels in patients with asthma were significantly associated with decreased lung function and enhanced eosinophilic inflammation. We identified five core target genes: CCR3, CCR7, CXCR4, IL2RA and IL2RB, among which CCR3 and IL2RA are key drivers of Th2 inflammation. CXCR4 and IL2RB are indirectly involved in the Th2 response through the modulation of cell migration and T cell activation, and together these genes constitute an immune regulatory network with a predominantly Th2-type inflammation. Further molecular docking showed that BPA stably binds to the above targets, and clinical trials have confirmed a correlation between the upregulation of expression of these genes and BPA in PBMCs from patients with asthma. These findings indicate that BPA may disrupt airway immune homeostasis by interfering with the JAK–STAT and chemokine signaling pathways and promoting a Th2 immune bias. This study provides preliminary evidence for the potential association between BPA exposure and asthma pathogenesis, and identifies core targets that may serve as candidate avenues for therapeutic intervention.

## Data Availability

The datasets presented in this study can be found in online repositories. The names of the repository/repositories and accession number(s) can be found below: https://www.ncbi.nlm.nih.gov/, GSE74968 https://www.ncbi.nlm.nih.gov/, GSE76262.
