# Supplementary material for: A mechanistic study of bisphenol a exposure on asthma through integrating clinical research, network toxicology, machine learning, and multi-omics
Source: Front Pharmacol. 2026 Jun 3;17:1861505. doi: 10.3389/fphar.2026.1861505 (PMC13272424; doi:10.3389/fphar.2026.1861505)

Table S1. Baseline characteristics of the total enrolled cohort and the qPCR subset in Patients with Asthma.

| **Variant** | **Total Asthma** | **qPCR Asthma** | **P-value** |
| --- | --- | --- | --- |
| Total (N) | 20 | 15 | / |
| Age, N (years) | 47.1±14.7 | 46.5±14.6 | 0.919 |
| Men (%) | 7 (35) | 6 (40) | 0.762 |
| BMI (kg/m^2^) | 23.0±4.2 | 23.4±2.6 | 0.750 |
| Ex-smokers, N (%) | 7 (35) | 5 (33) | 0.918 |
| Current smokers, N (%) | 3 (15) | 3 (20) | 0.698 |
| Smoking index (packets/year) | 0 (0, 6) | 0 (0, 6.5) | 0.987 |
| FEV1 predicted | 2.8 (2.6, 3.2) | 2.8 (2.6, 3.4) | 0.961 |
| FVC predicted | 3.4 (3.1, 3.7) | 3.3 (3.1, 4.3) | 0.934 |
| FEV1 (pre) | 2.1±0.9 | 2.1±0.9 | 0.911 |
| FVC (pre) | 3.2±1.0 | 3..1±1.0 | 0.898 |
| FEV1/FVC (%) (pre) | 64.0±13.2 | 63.9±13.9 | 0.977 |
| MEF75 (pre) | 2.9 (1.7, 5.4) | 2.8 (2.0, 5.8) | 0.882 |
| MEF25 (pre) | 0.5 (0.3, 1.1) | 0.5 (0.3, 1.3) | 0.987 |
| FEV1 (post) | 2.3±0.9 | 2.3±0.9 | 0.890 |
| FVC (post) | 3.3±0.9 | 3.3±0.9 | 0.912 |
| FEV1/FVC (%) (post) | 66.8±13.4 | 66.1±13.6 | 0.896 |
| MEF75 (post) | 3.9±2.2 | 3.9±2.4 | 0.986 |
| MEF25 (post) | 0.5 (0.3, 1.1) | 0.4 (0.3, 0.8) | 0.780 |
| FeNO (PPb) | 28.1±19.3 | 28.4±14.9 | 0.959 |
| Eosinophils (×10⁹/L) | 0.4±0.4 | 0.2±0.3 | 0.729 |
| Eosinophils (%) | 3.4 (1.7, 5.2) | 3.6 (2.4, 5.3) | 0.780 |
| Neutrophils (×10⁹/L) | 4.2±1.5 | 4.2±1.4 | 0.981 |
| Neutrophils (%) | 58.0±9.3 | 57.3±8.2 | 0.800 |
| Monocytes (×10⁹/L) | 0.4±0.1 | 0.4±0.2 | 0.841 |
| Monocytes (%) | 5.8±2.0 | 5.8±2.1 | 0.987 |
| Lymphocytes (×10⁹/L) | 2.3±0.7 | 2.5±0.7 | 0.427 |
| Lymphocytes (%) | 30.6±9.2 | 32.3±7.6 | 0.562 |
| Serum BPA level(ng/ml) | 3.2±1.5 | 3.2±1.6 | 0.985 |

BMI: body mass index; FEV1: first second expiratory volume with exertion; FVC: forced vital capacity; MEF: maximal expiratory flow. Data are expressed as mean±standard deviation or median (interquartile spacing) unless otherwise stated.

**Table S2.** Baseline characteristics of the total enrolled cohort and the qPCR subset in Healthy Controls.

| **Variant** | **Total Healthy Controls** | **qPCR Healthy Controls** | **P-value** |
| --- | --- | --- | --- |
| Total (N) | 10 | 9 | / |
| Age, N (years) | 43.7±13.8 | 42.3±15.1 | 0.957 |
| Men (%) | 4 (40) | 4 (40) | 0.845 |
| BMI (kg/m^2^) | 23.0±3.2 | 22.9±3.3 | 0.942 |
| Ex-smokers, N (%) | 3 (33.3) | 3 (33.3) | 0.876 |
| Current smokers, N (%) | 1 (10.0) | 1 (10.0) | 0.937 |
| Smoking index (packets/year) | 0 (0，6) | 0 (0, 6) | 0.905 |
| FEV1 predicted | 2.7 (2.4, 3.2) | 3.0 (2.3, 3.2) | 0.902 |
| FVC predicted | 3.2 (2.9, 3.6) | 3.4 (2.9, 3.7) | 0.842 |
| FEV1 (pre) | 2.8±0.4 | 2.8±0.4 | 0.799 |
| FVC (pre) | 3.5±0.4 | 3.5±0.3 | 0.736 |
| FEV1/FVC (%) (pre) | 82.0±4.8 | 82.4±4.9 | 0.870 |
| MEF75 (pre) | 5.8 (5.5, 6.2) | 6.0 (5.5, 6.2) | 0.905 |
| MEF25 (pre) | 1.5 (1.3, 2.0) | 1.5 (1.2, 2.1) | 0.905 |
| FEV1 (post) | 2.9±0.5 | 3.0±0.5 | 0.812 |
| FVC (post) | 3.6±0.8 | 3.6±0.9 | 0.871 |
| FEV1/FVC (%) (post) | 86.4±5.7 | 86.8±5.9 | 0.883 |
| MEF75 (post) | 6.8±1.4 | 6.8±1.5 | 0.981 |
| MEF25 (post) | 1.6 (0.9, 2.1) | 1.6 (10.0, 2.1) | 0.816 |
| FeNO (PPb) | 7.8±1.3 | 8.0±1.2 | 0.737 |
| Eosinophils (×10⁹/L) | 0.1±0.1 | 0.1±0.1 | 0.885 |
| Eosinophils (%) | 1.5 (1.0, 2.0) | 1.6 (0.9, 2.1) | 0.748 |
| Neutrophils (×10⁹/L) | 3.3±0.9 | 3.4±0.9 | 0.822 |
| Neutrophils (%) | 54.3±5.8 | 55.1±5.5 | 0.747 |
| Monocytes (×10⁹/L) | 0.4±0.7 | 0.4±0.1 | 0.893 |
| Monocytes (%) | 6.0±1.0 | 6.0±1.1 | 0.918 |
| Lymphocytes (×10⁹/L) | 2.3±1.3 | 2.3±0.7 | 0.991 |
| Lymphocytes (%) | 37.6±6.2 | 36.7±6.0 | 0.772 |
| Serum BPA level(ng/ml) | 1.3±0.4 | 1.4±0.4 | 0.950 |

BMI: body mass index; FEV1: first second expiratory volume with exertion; FVC: forced vital capacity; MEF: maximal expiratory flow. Data are expressed as mean±standard deviation or median (interquartile spacing) unless otherwise stated.

**Table S3.** Primer sequences for qPCR.

| **Gene** | **Forward primer (5'→3')** | **Reverse primer (5'→3')** | **bp** |
| --- | --- | --- | --- |
| GAPDH | gtcaaggctgagaacgggaa | aaatgagccccagccttctc | 158 |
| IL2RA | ggaagacaaggtggacccag | ttgtgacgaggcaggaagtc | 132 |
| IL2RB | gattttcagccaccccctga | ctggaccaggggaaactgac | 95 |
| CXCR4 | gctgttggctgaaaaggtgg | atctgcctcactgacgttgg | 97 |
| CCR3 | cgcctttgttggagagaggt | tagagagttccggctctgct | 155 |
| CCR7 | tggtgatcggctttctggtc | ccattgtagggcagctggaa | 160 |

bp:base pairs of the amplified product.

**Table S4.** Protein structures and docking grid parameters used in molecular docking

| **Targe**t | **UniProt ID** | **PDB ID** | **Method** | **Grid center (x, y, z)** |
| --- | --- | --- | --- | --- |
| IL2RA | P01589 | 1Z92 | X-ray | 14.048, -33.279, 12.454 |
| IL2RB | P14784 | 4GS7 | X-ray | 15.027, -29.215, 29.971 |
| CXCR4 | P61073 | 8yu7 | Cryo-EM | 149.332, 158.333, 130.904 |
| CCR3 | P51677 | 7x9y | Cryo-EM | 105.374, 128.181, 120.127 |
| CCR7 | P32248 | 6qzh | X-ray | -16.32, 7.104, -23.328 |

PDB, Protein Data Bank; Cryo-EM, cryo-electron microscopy. Grid spacing was set to 1.0 Å for all targets. Native ligands and water molecules were removed prior to docking.

**Figure S1** Venn diagrams of BPA and asthma-related target predictions and common genes. (A) BPA-associated targets from the ChEMBL, Swiss Target Prediction, and CTD databases. (B) Asthma targets from the GeneCards and OMIM databases. (C) Venn diagram showing predicted targets shared by BPA and asthma.


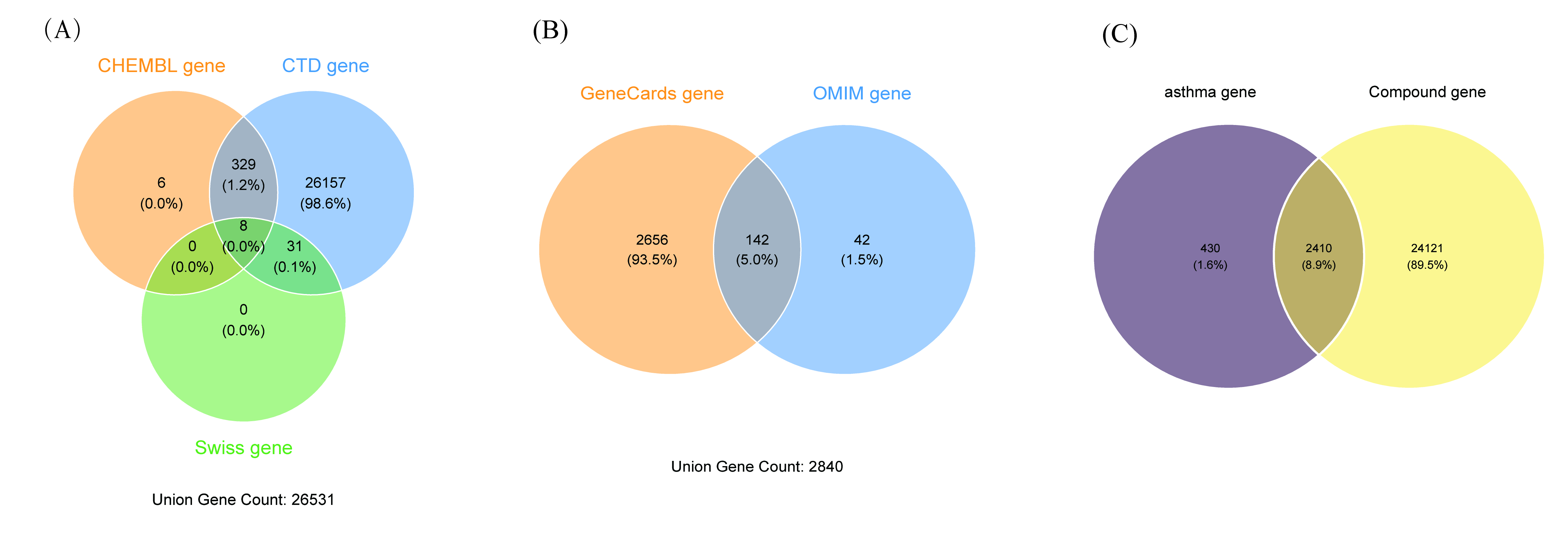


**Figure S2** (A) Volcano plot of the top 25 upregulated and downregulated genes based on logFC. (B) Venn plot showing BPA–asthma prediction of targets shared with differential genes. (C) Bubble plots of GO enrichment results.


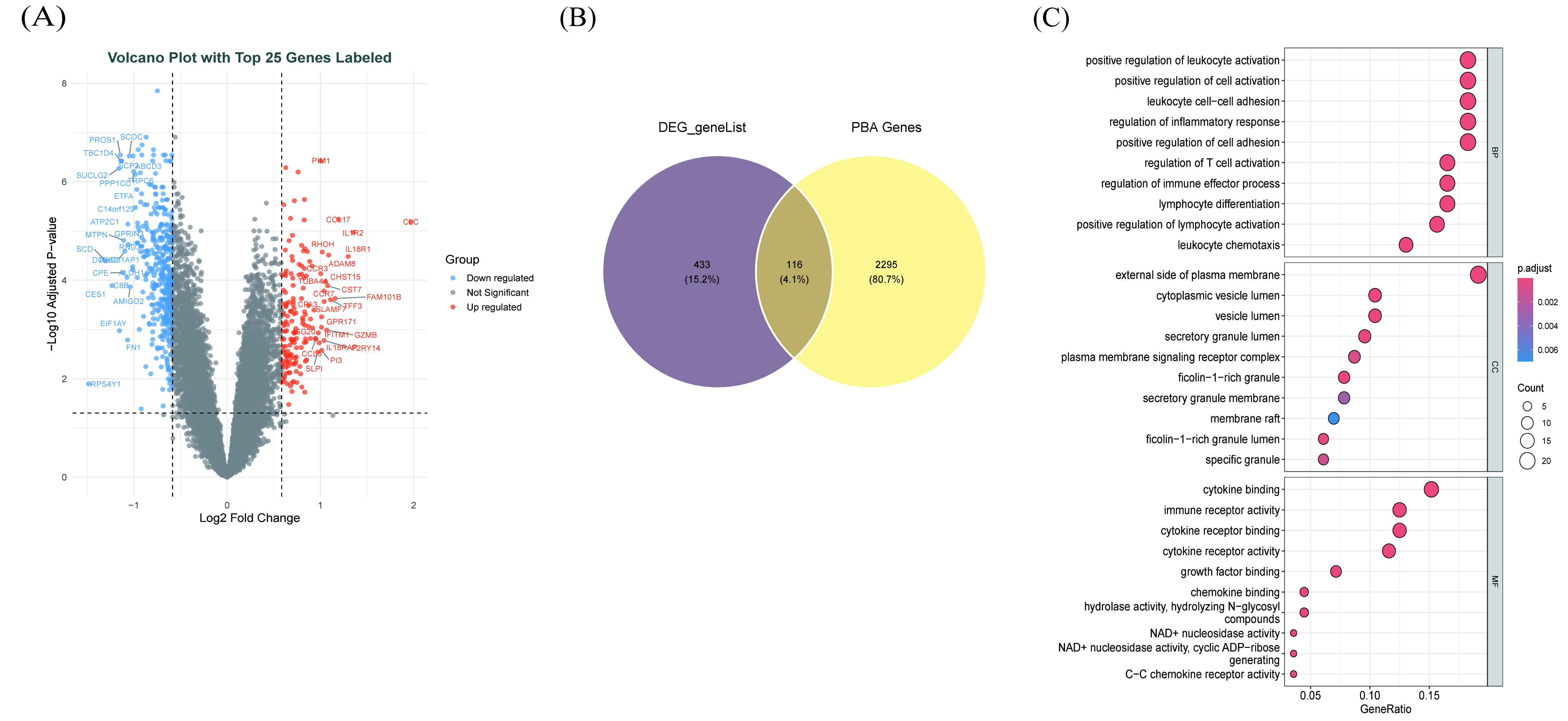


**Figure S3.** Correlation between serum BPA concentration and expression levels of candidate genes


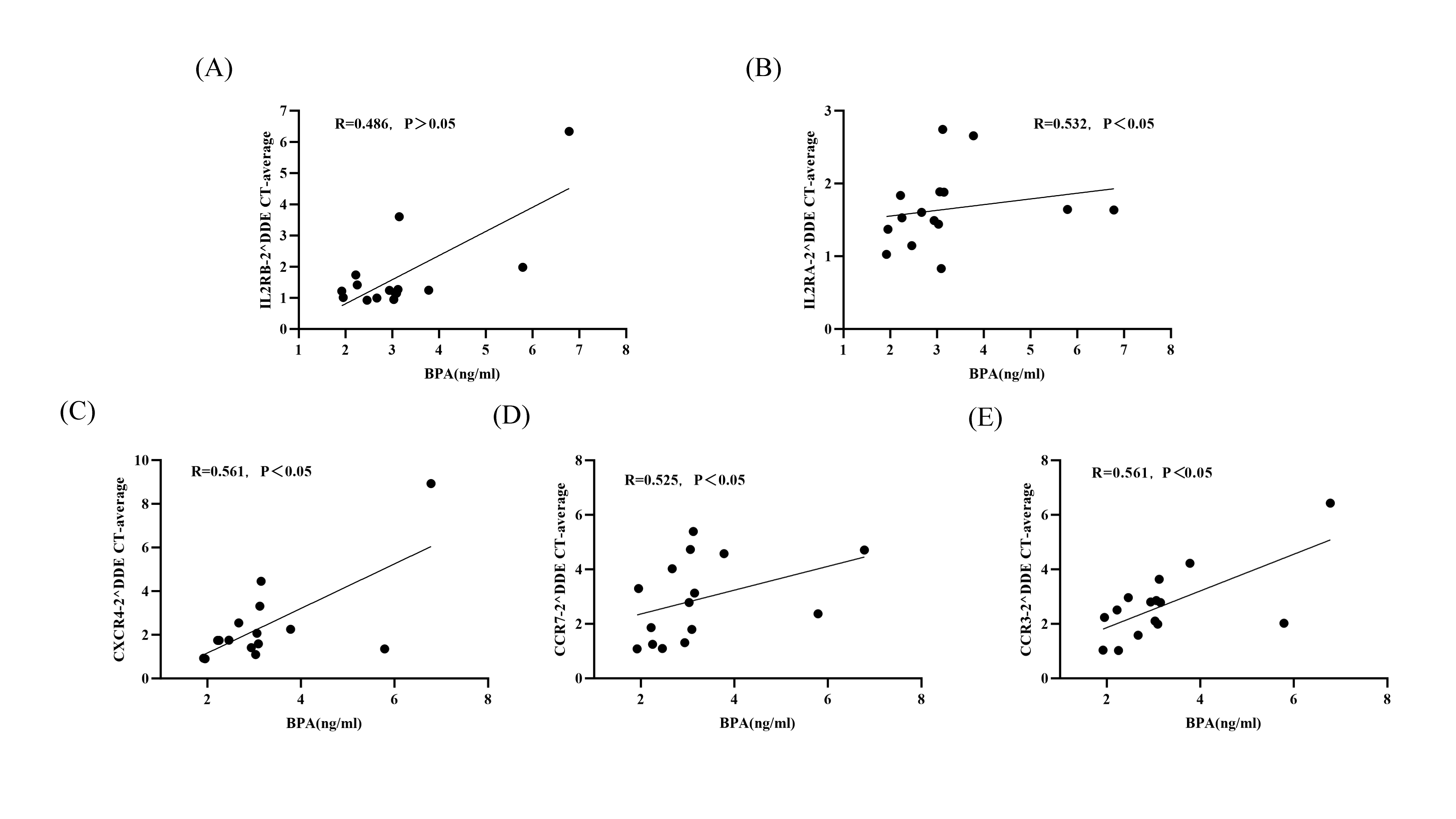

Supplement: Supplementary file 1 [file Supplementaryfile1.doc]
